# The Impact of Maternal Cigarette Smoke Exposure in a Rodent Model on Renal Development in the Offspring

**DOI:** 10.1371/journal.pone.0103443

**Published:** 2014-07-24

**Authors:** Ibrahim Al-Odat, Hui Chen, Yik Lung Chan, Sawiris Amgad, Muh Geot Wong, Anthony Gill, Carol Pollock, Sonia Saad

**Affiliations:** 1 School of Medical and Molecular Biosciences, The University of Technology Sydney, Ultimo, NSW, Australia; 2 Anatomical pathology, Northern Clinical School, St Leonards, NSW, Australia; 3 Renal Medicine, Kolling Institute, St Leonards, NSW, Australia; Universidade do Estado do Rio de Janeiro, Brazil

## Abstract

This study aimed to investigate whether maternal cigarette smoke exposure can disrupt fetal kidney development by changing the expression of growth and transcription factors essential for renal development, and thereafter predispose the offspring to chronic kidney disease later in life. Female Balb/c mice (6 weeks) were exposed either to cigarette smoke or air under identical conditions, 6 weeks prior to mating, during gestation and during lactation. Male offspring were sacrificed at three time points, postnatal day (P)1, P20 (weaning age), and 13 weeks (mature age). Blood, urine, and kidneys were collected for analysis. At P1, the developmental genes fibroblast growth factor 2, glial cell-line derived neurotrophic factor and paired box 2 were upregulated at mRNA and protein levels; whilst fibroblast growth factor (FGF) 7 and FGF10 were downregulated. At P20, mRNA expression of FGF2, FGF10 and Wingless-type 4 was upregulated by maternal smoke exposure. These changes were normalised in adulthood. Nephron development was delayed, with fewer nephron numbers from P1 persisted to adulthood; while glomerular volume was increased at P20 but reduced in adulthood. Pro-inflammatory marker monocyte chemoatractant protein 1 (MCP1) was increased in the kidney by maternal smoke exposure. These changes were accompanied by an increased albumin/creatinine ratio in adulthood, suggesting reduced renal dysfunction. In conclusion maternal cigarette smoke exposure prior to and during pregnancy, as well as lactation leads to significant renal underdevelopment and functional abnormalities in adulthood. This study confirms the hypothesis that maternal smoking predisposes offspring to chronic kidney disorders.

## Introduction

Many studies have established the association between maternal smoking and long-term health consequences in the offspring, including obesity, respiratory and cardiovascular diseases [1], [2]. Maternal smoking is associated with intrauterine growth retardation (IUGR); while IUGR in turn, is associated with reduced nephron number in the offspring [3]. Indeed, human research has shown that maternal smoking is closely linked to lower fetal kidney volumes during the second and third trimester, and lower birth weight [4]. The reduction in fetal kidney volume correlates with the number of cigarettes smoked in a dose-dependent manner. Only maternal smoking greater than 10 cigarettes/day led to smaller fetal kidney volumes [5].

In humans, nephrogenesis starts at gestational week 6–8 [6], and ends at gestational week 36 [7]. Most nephrons are formed in the third trimester [8], and the final number of nephrons in each kidney is established at birth. However, in rodents, nephrogenesis continues after birth for a short period of time until weaning [9]. Kidney development is regulated by a highly coordinated activation of multiple growth factors and transcriptional regulators [10]. Alteration of these growth factors at any stage of kidney development can lead to renal underdevelopment and potential renal dysfunction in the long term [11], [12]. Few studies have directly investigated the mechanisms underlying renal developmental abnormalities induced by maternal smoking, and the results from available literature are contradictory. Animal studies have shown both increased [13], and unchanged apoptosis [14] in the offspring kidney of smoke exposed dams. One study using proteomic analyses in mice did not observe changes in protein expression of factors involved in renal growth, albeit small birth weight and kidney mass [15]. However, it could be argued that the cigarette smoke dose in this model was greater than expected in human correlates [15]. Maternal smoking may impair renal function in the offspring once adulthood is reached [5], [16], [17], due to abnormal early development, including low numbers of nephrons and secondary hyperfiltration [5]. However, it is still unknown how maternal smoking is linked to an increased risk of developing chronic kidney disease in offspring [18].

This study aimed to investigate the changes of kidney structure and factors that regulate renal development at different postnatal ages, including birth, weaning and adulthood, as well as renal function in adulthood, in mice offspring of dams exposed to cigarette smoke before and during gestation and lactation.

## Materials and Methods

### 1. Ethics Statement

The animal experiment was approved by the Animal Care and Ethics Committee at the University of Technology, Sydney (ACEC#2011-313A). All protocols were performed according to the Australian NH&MRC Guide for the Care and Use of Laboratory Animals.

### 2. Animal and tissue collection

Female Balb/c mice (6 weeks, n = 40, Animal Resources Centre, Perth, Australia) were housed at 20±2°C and maintained on a 12∶12 hour light/dark cycle (lights on 06∶00 h). After acclimatization, the mice were divided into 2 groups with equal body weight (BW), the sham exposed (control, n = 20) and cigarette smoke exposed (SE, n = 20) groups. Animals in the SE group were placed inside a perspex chamber (18 liters) filled up with the smoke produced by two cigarettes (Winfield Red, 16 mg or less of tar, 1.2 mg or less of nicotine and 15 mg or less of CO; Philip Morris, Melbourne, Australia) a time with a 5-minute interval between, twice (10∶00 and 15∶30) daily. Control mice were exposed to air in an identical chamber at the same time as previously described [19], [20]. Six weeks later, females were mated. Cigarette smoke exposure continued throughout gestation and lactation periods. The sires and offspring were not exposed. Pups were weaned at postnatal day (P)20 and maintained without additional intervention. The dams were culled when the pups weaned.

Male offspring were scarified at P1, P20 (weaning age) and week 13 (W13, mature age) (n = 11–23). They were weighed and anaesthetized with sodium thiopental (0.1 ml/g, i.p., Abbott Australasia PTY. LTD, NSW, Australia). Blood was collected through cardiac puncture and blood glucose was measured (Accu-chek, Roche Diagnostics, Nutley, USA). Mice were killed by decapitation. The left kidneys were fixed with 10% formalin (Sigma, VIC, Australia); the right kidneys were snap frozen in liquid nitrogen and stored at −80°C. Urine was collected from the bladder when available.

### 3. Intraperitoneal glucose tolerance test (IPGTT)

IPGTT was performed at 12 weeks as previously described [20]. Briefly, after 5 h fasting, a baseline glucose level was taken from tail blood (Accu-Chek, Roche Diagnostics, Indianapolis, IN, USA). Glucose was then administered (2 g/kg i.p., *n* = 9–10) and blood glucose levels were measured at 15, 30, 60, and 90 min post-injection [20]. The area under the curve (AUC) of the glucose levels was calculated for each mouse.

### 4. Real time (rt) PCR

Total RNA was extracted from the kidney using Trizol (Life Technology, CA, USA). First strand cDNA was generated using Transcriptor First Strand cDNA Synthesis Kit (Roche Diagnostics, Mannheim, Germany). rt-PCR was performed using pre-optimized SYBR Green primers (Sigma-Aldrich, Table 1) and rt-PCR master mix (Life Technology, CA, USA) as previously described [21]. The average mRNA expression of the Control group was used as the calibrator and 18S rRNA was used as the housekeeping gene [21].

**Table 1:** Forward and reverse sequences of the primers for rt-PCR.

Primer	Forward 5′-3′	Reveres 5′-3′
**18S**	CGCGGTTCTATTTTGTTGGT	AGTCGGCATCGTTTATGGTC
**BMP7**	CACTCCCTCCTCAACCCTCGGCA	TAGAGGCATCATAGGCCAGGTGCCC
**BMP4**	GGTCCAGGAAGAAGAATAA	GGTACAACATGGAAATGG
**Collagen IV**	TTAAAGGACTCCAGGGACCAC	CCCACTGAGCCTGTCACAC
**FGF2**	GACCCCAAGCGGCTCTACTGC	GTGCCACATACCAACTGGAGT
**FGF7**	GGCAATCAAAGGGGTGGA	CCTCCG CTG TGTGTCCATTTA
**FGF10**	TGAGACAATTTCCAGTGCCG	TATCTCCAGGACACTGTACG
**FN**	CACGGAGGCCACCATTACT	CTTCAGGGCAATGACGTAGAT
**GDNF**	ATTTTATTCAAGCCACCATTA	GATACATCCACACCGTTTAGC
**MCP1**	CATCCACGTGTTGGCTCA	GATCATCTTGCTGGTGAATGAGT
**Pax2**	CGCCGTTTCTGTGACACACAATC	TGCTTGGGACCAAACACAAGGTG
**WNT4**	AGGAGTGCCAATACCAGTTCC	TGTGAGAAGGCTACGCCATA
**WNT11**	CAGGATCCCAAGCCAATAAA	GACAGGTAGCGGGTCTTGAG
**WT1**	ATCAGATGAACCTAGGAG	CTGGGTATGCACACATGA

### 5. Kidney histology and immunohistochemistry (IHC) staining

Fixed kidney samples were embedded in paraffin and sectioned. Kidney structure of the offspring was examined using hematoxylin and eosin (H&E) and periodic acid Schiff stain (PAS).

For IHC staining, kidney sections were incubated with primary antibodies against FGF2, GDNF (Santa Cruz Biotechnology, CA, USA) and Pax 2 (Abcam, Cambridge, UK). The tissues were then incubated with polymer secondary anti-rabbit antibodies (Dako Ref K4003) and horseradish peroxidise enzyme and DAB+ (liquid DAB+substrate chromogen system, Dako Ref K3468). The section were counterstaining with haematoxylin. Negative controls were prepared by replacing the primary antibodies with rabbit IgG. Quantitation of the positive signals in the images was performed using Image J software (Image J, NIH, USA).

Glomerular number was estimated by counting the developed glomeruli in 8–10 different fields for the same kidney section then averaged. One random kidney section was used from 3–4 different biological repeats from each group. Glomerular size was measured using Image J (Image J, NIH, USA) in 8–10 different images for the same kidney section then averaged. Glomerular and tubular structure, in addition to glomerular number and size were additionally assessed by an independent pathologist in a blinded manner for confirmation.

### 6. Albumin and creatinine assays

Urine albumin and creatinine were measured using Murine Microalbuminuria ELISA kit (Albuwell M) and Creatinine Companion kit, respectively (Exocell Inc, PA, USA). Serum enzymatic creatinine levels were measured by an automated analyzer (ARCHITECT, Abbott Australasia, Australia).

### 7. Statistical analysis

The differences between the groups were analyzed using unpaired Student’s *t-*test (Prism 6, Graphpad CA, USA). The results are expressed as mean±SEM. P<0.05 is considered significant.

## Results

### 1. Female breeders

Before the commencement of smoke exposure, the BW was similar between the Control and SE groups (Table 2). Six weeks later, the SE dams had significantly smaller BW compared with the Control dams (p<0.05, Table 2). At the endpoint, BW, kidney weight and liver weight were significantly lower in the SE dams (p<0.01). Blood glucose levels were similar between groups (Table 2).

**Table 2:** The effects of smoking on female breeders.

Breeders	Control	SE
Body weight before smoke exposure (g)	17.6±0.2	17.4±0.2
Body weight at mating (g)	19.2±0.2	17.6±0.2**
Body weight at cull (g)	24.6±0.4	21.9±0.2**
Kidney (g)	0.17±0.003	0.15±0.003**
Kidney%	0.69±0.01	0.66±0.04
Liver (g)	1.78±0.05	1.44±0.04**
Liver%	7.22±0.12	6.53±0.15**
Blood glucose (mM)	12.4±1.1	10.6±0.8

Results are expressed as mean ± SEM, n = 20.

*p<0.05,

**p<0.01.

SE: smoke exposed.

### 2. Parameters in the offspring

**Figure 1:**
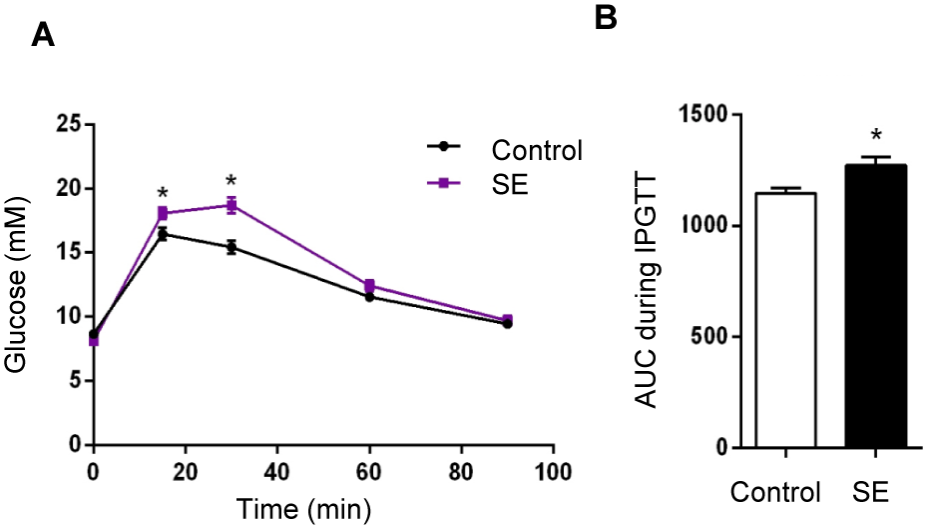
Glucose tolerance test in offspring of SE dams. (A) Blood glucose changes during IPGTT over time. (B) Area under the curve of (A) showing that offspring from SE dams are glucose intolerant at adulthood (W13). n = 9–10. *p<0.05, maternal smoke exposure effect.

At P1 and P20, the BW and organ weights of the SE offspring were similar to the Controls (Table 3). At W13, BW and kidney weight were still significantly lower in the SE offspring (p<0.05, Table 3). There was no difference in blood glucose levels at all ages (Table 3). However, IPGTT showed a significantly increased blood glucose level at 15 and 30 min post-injection (p<0.05, Figure 1A). AUC also showed significant glucose intolerance (Figure 1B) in the SE offspring compare to the Control (p<0.05).

**Table 3:** The effects of maternal smoke exposure on growth and development in offspring.

Male offspring	P1		P20		W13	
	Control	SE	Control	SE	Control	SE
Body weight (g)	1.48±0.05	1.51±0.07	9.90±0.22	9.70±0.22	26.1±0.4	24.6±0.4*
Kidney (g)	0.01±0.001	0.01±0.001	0.07±0.002	0.07±0.002	0.22±0.003	0.19±0.005*
Kidney%	0.67±0.11	0.60±0.09	0.70±0.01	0.71±0.01	0.85±0.01	0.78±0.01*
Liver (g)	0.04±0.01	0.04±0.01	0.43±0.01	0.42±0.02	1.30±0.02	1.27±0.03
Liver%	2.92±0.3	2.83±0.5	4.38±0.06	4.32±0.09	4.99±0.05	5.15±0.07
Glucose (mM)	4.70±0.2	4.23±0.1	10.8±0.5	11.4±0.5	10.8±0.4	10.1±0.3

Results are expressed as mean ± SEM, n = 11–23.

*p<0.05.

SE: smoke exposed.

### 3. Kidney histological changes and glomerular numbers

**Figure 2:**
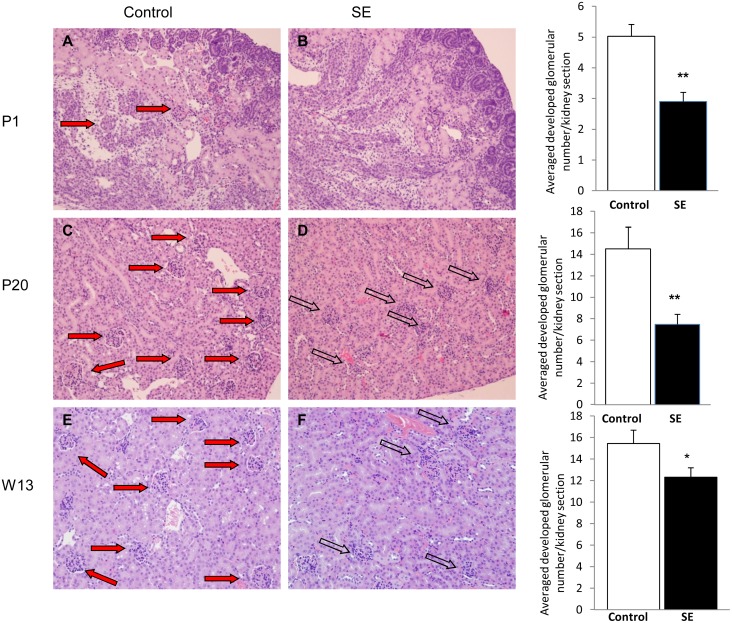
Glomerular number in offspring of SE dams. Kidney H&E stained sections from the offspring of Control (1^st^ column) and smoke exposed dams (SE, 2^nd^ column right panel) at postnatal (P)1 (A, B), P20 (C, D), and week (W)13 (E, F). Reduced glomerular numbers was shown in offspring from SE dams at birth (P1), early postnatal life (P20) and adulthood (W13). Mag. 20X. Closed arrows show mature and fully vascularized glomeruli and open arrows show underdeveloped glomeruli. Numbers of developed glomeruli are shown in the 3^rd^ column. n = 6–8. *p<0.05 and #p<0.01, maternal smoke exposure effect.

**Figure 3:**
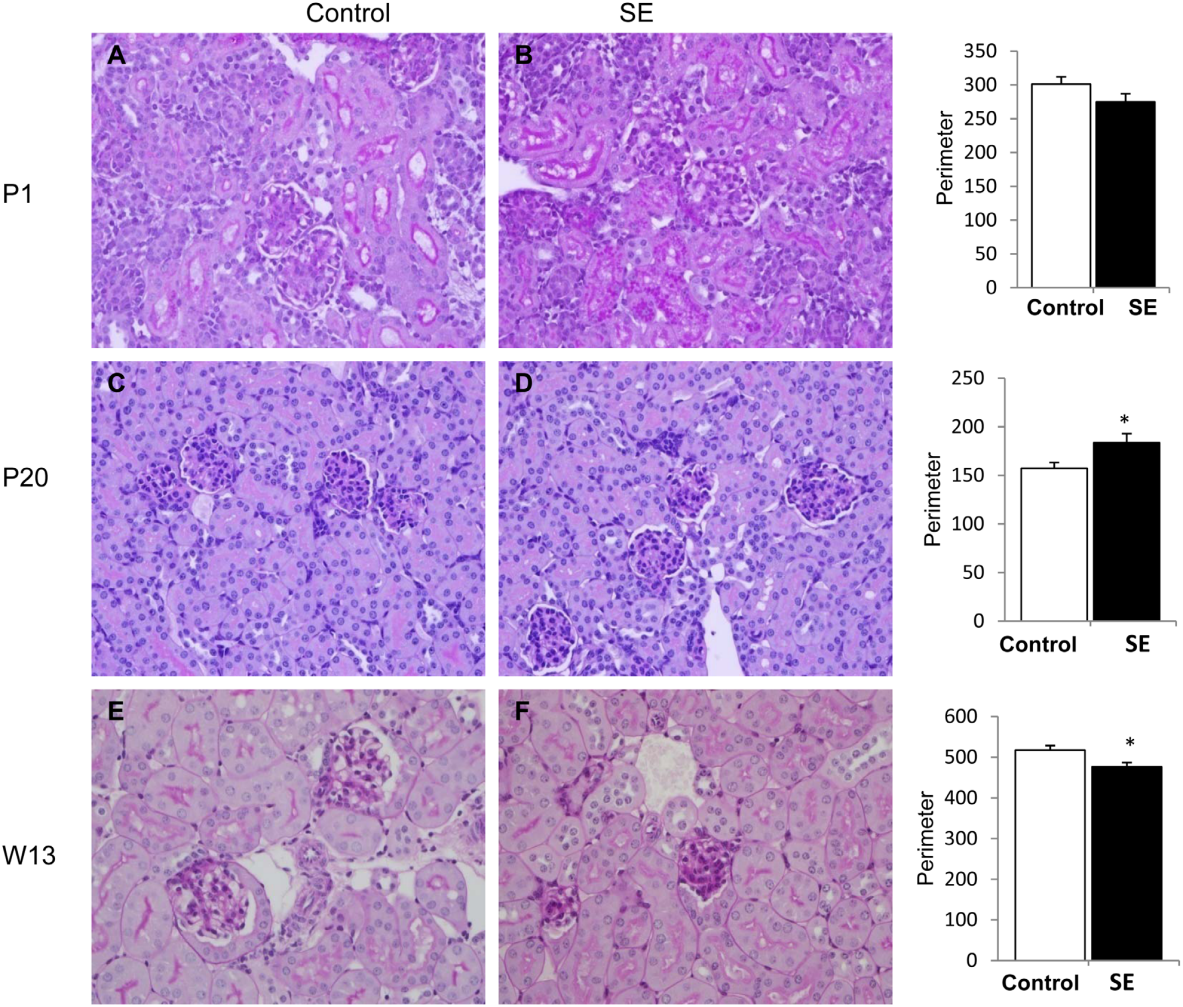
Glomeruli size in offspring of SE dams. Kidney PAS staining showing glomerular size in the offspring of Control (1^st^ column) and smoke exposed dams (SE, 2^nd^ column) at postnatal (P)1 (A, B), P20 (C, D) and week (W)13 (E, F). Enlarged glomeruli are shown in early postnatal life (P20) and reduced glomeruli size was shown at adulthood (W13) in offspring from SE dams. n = 3–4. *p<0.05 maternal smoke exposure effect.

At P1, the SE offspring had fewer glomeruli and more immature (non-vascularized) glomeruli compared with the Control offspring (p<0.01, Figure 2A, B). At P20, fewer developed and more underdeveloped glomeruli were still evident in the SE offspring compared to the Control offspring (p<0.01, Figure 2C, D). At W13, SE offspring still got smaller glomerular number than the Control offspring (P<0.05), although the glomeruli were structurally developed in the adult SE offspring (Figure 2E, F). The glomerular size in the SE offspring was similar to the Control offspring at P1; at P20, the mature glomerular size in the SE offspring was significantly larger than the Control offspring (p<0.05, Figure 3). However, by W13 glomerular size was significantly decreased in the SE offspring compared with the Control (p<0.05, Figure 3). In the offspring of SE dams, some glomeruli had minor degrees (<25%) of sclerosis, and some tubules were elongated with proteinacious material. However, there was no statistical significant on these changes (data not shown).

### 4. Kidney mRNA and protein levels

**Figure 4:**
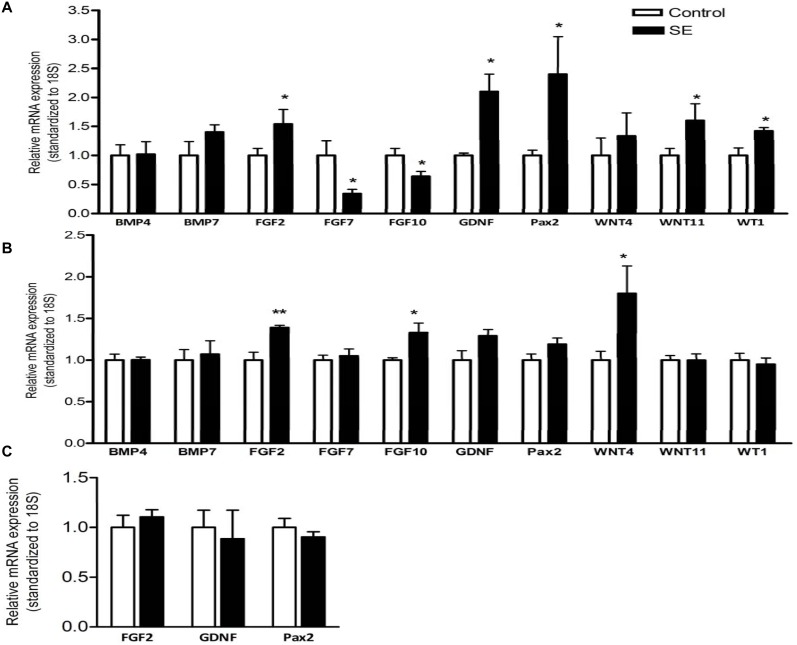
Renal growth and transcription factors mRNA expression in offspring mice. Renal mRNA expression of growth factors in the offspring at postnatal (P)1 (A), P20 (B) and week (W)13 (C) showing increased expression of FGF2, GDNF and Pax2 at birth and early postnatal life (P1 and P20 respectively). n = 3–6. *p<0.05, **P<0.01 vs maternal smoke exposure effect. BMP: Bone morphogenetic proteins; GDNF: glial-cell line-derived neurotrophic factor; FGF: fibroblast growth factors; Pax: paired box transcription factor; WNT: wingless–type MMTV integration site family members; WT: Wilms tumour inhibitory protein.

Maternal smoke exposure differentially affected mRNA expression of the growth and transcription factors involved in the renal development. Renal mRNA expression of fibroblast growth factors (FGF)2, glial-cell line-derived neurotrophic factor (GDNF), paired box transcription factor (Pax)2, wingless–type MMTV integration site family members (WNT)11 and Wilms tumour inhibitory protein (WT)1 was significantly upregulated, whilst FGF7 and FGF10 were downregulated by maternal smoke exposure at P1 (p<0.05, Figure 4A). Renal bone morphogenetic proteins (BMP)4, BMP7, WNT4 mRNA expression were not changed at this age. At P20, renal mRNA expression of FGF2, FGF10 and WNT4 was significantly upregulated by maternal SE (p<0.01, FGF2; p<0.05, FGF10, WNT4, Figure 4B). However, mRNA levels of renal developmental genes were not different between groups in adulthood when renal development has completed (Figure 4C).

**Figure 5:**
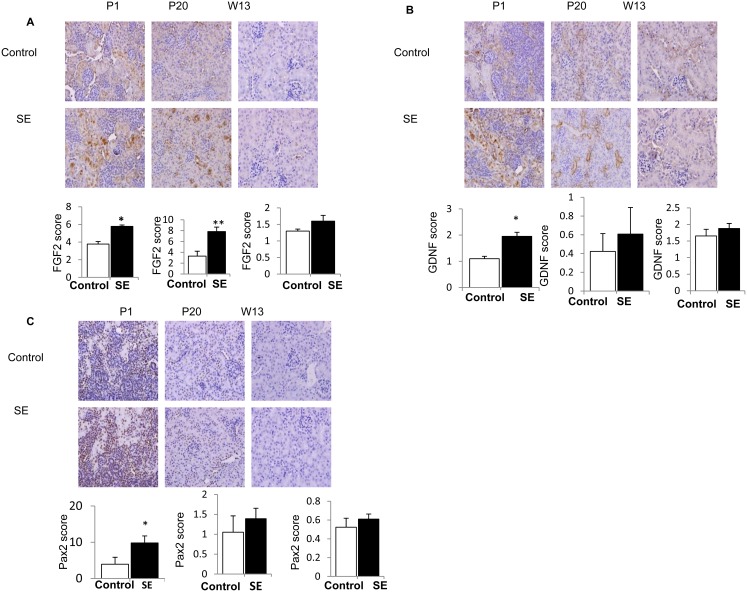
Renal growth and transcription factors protein expression in offspring mice. (A) Immunostaining showing increased protein expression of fibroblast growth factors (FGF2) at birth and early postnatal life (P1 and P20 respectively). (B) Increased protein expression of Glial-cell line-derived neurotrophic factor protein expression (GDNF) at birth and (C) increased protein expression of Paired box transcription factor (Pax2) in offspring from SE dams compare to offspring from control dams. Mag. 40X. n = 3–4. *p<0.05, maternal smoke exposure effect.

Renal protein levels of FGF2, GDNF and Pax2 were higher in the offspring of SE dams at P1 (p<0.05, Figure 5A, B, C). FGF2 protein level was still higher at P20 in the SE offspring, but not at W13; while GDNF and Pax2 protein levels were not different between groups at P20 and W13 (Figure 5A, B, C).

### 5. Renal markers of inflammation, injury and function

**Figure 6:**
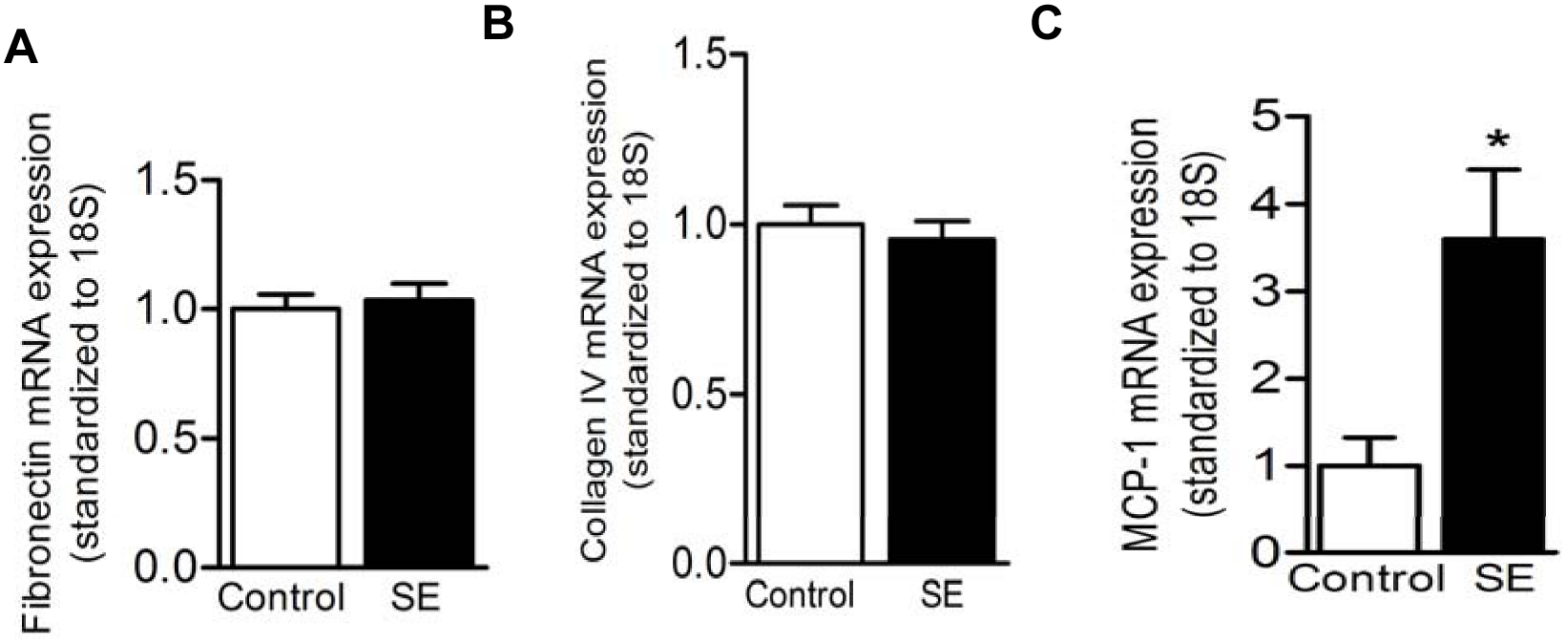
Expression of kidney injury markers in offspring mice at W13. Renal mRNA expression of pro-fibrotic marker fibronectin (A) and collagen IV (B) did not change between the offspring from SE dams and control dams due to maternal smoke exposure at week (W)13. Pro-inflammatory marker MCP-1 (C) mRNA expression in offspring from SE dams was significantly higher compare to offspring from control dams at W13. n = 4–6. *p<0.05, maternal smoke exposure effect.

mRNA levels of pro-fibrotic markers (fibronectin and collagen IV) and pro-inflammatory marker (MCP-1) were measured in the offspring kidney at adulthood. There was no difference between fibronectin and collagen IV mRNA levels at W13 (Figure 6A, B). However, MCP-1 mRNA expression was significantly increased in the SE offspring (p<0.05, Figure 6A). This was accompanied by increased urinary albumin/creatinine ratio at W13 (p<0.05, Table 4). There were also no differences in the serum enzymatic creatinine levels at all ages (Table 4).

**Table 4:** The effects of maternal smoke exposure on renal function in offspring.

Male offspring	P20		W13	
	Control	SE	Control	SE
Urinary albumin/creatinine ratio (µg/mg)	17.0±4.8	6.0±2.4	7.00±2.3	38.0±6.3*
Serum enzymatic creatinine (µmol/l)	10.4±0.7	12.1±1.2	15.2±1.3	14.2±0.5

Results are expressed as mean ± SEM, n = 3–11.

*p<0.05.

SE: smoke exposed.

## Discussion

The major finding of the current study is that maternal cigarette smoke exposure from pre-gestation to lactation period is clearly associated with abnormal early kidney development in offspring and resulting renal dysfunction and increased inflammatory markers in adulthood.

After 6 weeks of cigarette smoke exposure, smaller body weight was observed in the SE dams, which is consistent with our previous study [22]. Maternal smoking is known to cause intrauterine growth retardation and resultant small fetal kidneys in humans [3]. We have demonstrated that offspring from SE dams have reduced nephron numbers at birth and weaning but no difference in kidney weight, which is consistent with previous animal studies [13], [23]. This is not surprising especially since body weight and kidney weight don’t correlate with nephron number [24]. In animal studies, only suprapharmacologically high dose of cigarette smoke have been shown to cause significantly smaller kidney size in the offspring [15]. In this study, male SE offspring displayed smaller body weight, and kidney mass in mature age (13 weeks), which was opposite to our previous observation [20]. This may be due to gender difference as only females were reported in our previous study. However, glucose intolerance developed at 12 weeks in the SE offspring was consistent with our previous study in the female offspring [20]. We have shown that TNFα mRNA expression was upregulated in the fat tissue, which may play an important role in systemic glucose intolerance in the glucose deposit organs, such as fat itself and skeletal muscle [20]. In the liver, insulin sensitivity did not seem to be affected by maternal cigarette smoke exposure in the females [20]. Whether the same occurs in the male offspring requires further investigation, which is beyond the scope of the current study. In addition, the female offspring in our previous study were heavier with more adiposity than the control offspring. This may be due to the difference in the fat concentration in the diet, which was 14% in the previous study [20] versus 11% in the current study. It has been suggested that the offspring of the smokers are more likely to develop obesity due to their preference for junk diet [25]. In addition, gender difference may also need to be taken into consideration. Nevertheless, it also suggests that the metabolic disorder induced by maternal SE can be independent of postnatal body weight. Diabetes is one of the risk factors to develop kidney functional disorders and smokers’ offspring are predisposed to diabetes in human studies (reviewed in [25]). In addition, in this study, renal underdevelopment at early postnatal life and renal dysfunction in adulthood in the SE offspring is consistent with previous human studies [5], suggesting the close association between our mouse model and human conditions. As such, it cannot be excluded that glucose intolerance in the SE offspring may also contribute their already developed kidney dysfunction at 13 weeks, rendering them to possible chronic kidney disease later in life.

During kidney development, environmental factors can affect the nephron numbers during nephrogenesis. Maternal smoking, particularly in the first trimester, has been shown to impose a significant adverse impact on fetal renal development and the future risk of chronic kidney disease [26], [27]. However, it has been reported that the correlations between maternal smoking and abnormal renal mass disappeared by age [5], potentially due to ‘catch up’ growth commonly seen in such offspring [2]. In this study, the delayed renal development observed immediately after birth and at weaning led to fewer and smaller mature glomeruli in the adulthood in the SE offspring, regardless of the kidney mass, suggesting the importance of early renal development in determining future renal function.

There are several proposed mechanisms that may contribute to fetal renal underdevelopment [28]. Changes in the growth factors in the uterus can clearly play an important role. FGF7 and FGF10 are essential to stimulate the proliferation, migration and differentiation of epithelial cells at the growing tips of the ureteric bud [29], [30]. Therefore, FGF7 and FGF10 may also determine the number of mature nephron [31]. In this study, mRNA expression of FGF7 and FGF10 were lower at P1 in the SE offspring, which may directly lead to reduced ureteric bud growth and branching resulting in reduced numbers of glomeruli after birth. However at P20, FGF7 and FGF10 mRNA expression was either normalized or upregulated, which may promote the catch-up growth of kidney development and maturation after weaning. Indeed, at P20 although glomeruli number was still reduced by maternal smoking, the glomeruli size was significantly increased in the SE offspring suggesting a compensatory effect of the reduced numbers of glomeruli in order to maintain necessary renal function [26].

In this study, adaptations of other growth factors have also been observed in the SE offspring. The GDNF signaling pathway is critical for the initial stage of nephrogenesis [6], [9], [32], [33], [34], [35], [36], [37], [38]. GDNF is expressed by metanephric mesenchyme, which induces the ureteric bud outgrowth and branching. Disruption of the GDNF pathway has been shown to cause low nephron number due to its critical involvement in the initiation of nephrogenesis [39]. Fibroblast growth factor, FGF2 promotes ureteric bud endothelial cell proliferation to form collecting ducts [31], [38]. Here, offspring from SE dams have increased GDNF and FGF2 mRNA and protein levels at P1 and P20, suggesting early adaptation to intrauterine renal underdevelopment. Pax2 is expressed at the ureteric bud and metanephric mesenchyme in the developing kidneys, which is essential for developing renal epithelium and generating tubules from the mesenchyme [40]. WT1 is involved in metanephric cell differentiation into epithelial cells [41]. Pax2 induces WT1 expression in the metanephric mesenchyme which acts as a negative feedback for Pax2 expression when the metanehpric mesenchyme has been differentiated into epithelial cells [42], [43]. Interestingly Pax2 mRNA and protein expression at P1 and P20 are positively correlated with WT1 expression. During glomerular formation, WNT 4 is a autocrine signal to promote the condensation and the aggregation of the metanephric mesenchyme around the tip of the T-shape ureteric bud to form glomeruli [38]. Interestingly, WNT4 is increased at P20 but its level was not changed at P1, suggesting a possible role of WNT4 to increase in glomerular size at P20. As expected, the basal levels of all the growth factors were low or undetectable at W13, most likely due to the completion of nephrogenesis.

WT1, BMP7, BMP4 and Pax2 are all anti-apoptotic factors [41] which function at different stages of renal development. During fetal development, apoptosis regulates ureteric budding, which is essential to determine nephron numbers [39]. Placental insufficiency can promote cellular apoptosis resulting in reduced nephron number [39]. Indeed, increased renal apoptosis has been reported in offspring of SE dams [13]. In the current study, the upregulation of WT1 at P1 by maternal smoke exposure may be an adaptation to reduce apoptosis and to increase nephrogenesis. BMP7 is expressed in the ureteric bud and cap mesenchyme to induce ureteric budding [38], [41], while BMP4 can prevent ectopic budding [44]. Therefore, they are more likely to change during intrauterine fetal development, but not after birth [45].

A previous study [15] in the offspring of cigarette smoke exposed mice used proteomics to show subsequent changes in the expression of renal proteins that regulate inflammation, cell to cell signaling/interactions, lipid metabolism, small molecule biochemistry, cell cycle, nucleic acid and carbohydrate metabolism network. However, it did not address any of the growth factors involved in renal development. Short term high dose cigarette smoke exposure has been shown to increase oxidative stress in the mice kidney, which is one of the suggestive mechanisms leading to both chemical induced underdevelopment of fetal kidney and the onset of chronic kidney disease in adults [46], [47], [48]. However, whether this is involved in maternal smoking related renal underdevelopment in offspring requires further investigation. Regardless, chemicals in the cigarette smoke are known to cause direct damage to the smokers’ kidney, leading to proximal tubular damage, kidney cancer, and end-stage kidney disease [49]. The chemicals, such as nicotine, inhaled by the pregnant mothers, pass rapidly across the placenta and accumulate in the kidney, which has been shown to lead to smaller kidneys in offspring from rat dams with nicotine infusion [50]. The exposure of other chemicals in the cigarette smoke, such as cadmium and polycyclic aromatic hydrocarbons, have been shown to be correlated with the risk of fetal growth restriction, however with the direct impact on kidney development unknown [50]. As tobacco smoke is contains more than 4000 chemical substances [51], using direct cigarette smoke expose will re-produce a better effect of such intrauterine factor in animal studies.

Here we show that underdevelopment of the kidney in the offspring of SE dams is closely associated with abnormal renal function, reflected by increased urinary albumin/creatinine excretion. Proteinuria is a biomarker reflecting progressive renal dysfunction [52]. It has been suggested that reduced nephron number at birth can lead to the adaptation of glomerular hypertrophy and hyperfiltration in order to maintain sufficient renal function [26], [28], [39], [53]. Prolonged hyperfiltration can in turn lead to hyperfiltration injury in the long term, resulting in structural injury and functional deterioration in adulthood [26].

Structurally, subtle changes were observed in the tubules and glomeruli in the kidneys of the SE offspring at W13. However, the expression of the pro-fibrotic markers, fibronectin and collagen IV were not altered, maybe due to the sensitivity of the methodology or relatively young age of the mice. Inflammation correlates with renal damage and contributes to the development of chronic kidney disease [54]. MCP-1 is a pro-inflammatory and pro-fibrotic protein [54]. In recent studies, it has been demonstrated that MCP-1 is involved in the initiation and progression of glomerular and tubulointerstitial damage [52], [55]. Moreover, MCP-1 expression is also positively correlated with albuminuria [55], which was well presented in this study. As a result, the offspring of SE dams may be prone to further renal damage as adulthood progresses.

## Conclusion

In this study, maternal smoke exposure leads to significant developmental abnormalities in the kidney in early life and functional deterioration in adulthood. Changes in the expression of growth factors in early life and pro-inflammatory markers are a key mechanism underpinning abnormal renal development and the future risk of chronic kidney disease.
